# Gallstones, cholecystectomy and risk of cancers of the liver, biliary tract and pancreas

**DOI:** 10.1038/sj.bjc.6690101

**Published:** 1999-02

**Authors:** W H Chow, C Johansen, G Gridley, L Mellemkjær, J H Olsen, J F Fraumeni Jr

**Affiliations:** Division of Cancer Epidemiology and Genetics, National Cancer Institute, Bethesda, MD, USA; Danish Cancer Society, Institute of Cancer Epidemiology, Copenhagen, Denmark

**Keywords:** gallstones, cholecystectomy, neoplasms, liver, biliary tract, pancreas, cohort study

## Abstract

To examine the association between gallstones and cholecystectomy, we conducted a nationwide population-based cohort study in Denmark. Patients with a discharge diagnosis of gallstones from 1977 to 1989 were identified from the Danish National Registry of Patients and followed up for cancer occurrence until death or the end of 1993 by record linkage to the Danish Cancer Registry. Included in the cohort were 60 176 patients, with 471 450 person–years of follow-up. Cancer risks were estimated by standardized incidence ratios (SIRs) and 95% confidence intervals (CIs) stratified by years of follow-up and by cholecystectomy status. Among patients without cholecystectomy, the risks at 5 or more years of follow-up were significantly elevated for cancers of liver (SIR = 2.0, CI = 1.2–3.1) and gallbladder (SIR = 2.7, CI = 1.5–4.4) and near unity for cancers of extrahepatic bile duct (SIR = 1.1), ampulla of Vater (SIR = 1.0) and pancreas (SIR = 1.1). The excess risk of liver cancer was seen only among patients with a history of hepatic disease. Among cholecystectomy patients, the risks at 5 or more years of follow-up declined for cancers of liver (SIR = 1.1) and extrahepatic bile duct (SIR = 0.7), but were elevated for cancers of ampulla of Vater (SIR = 2.0, CI = 1.0–3.7) and pancreas (SIR = 1.3, CI = 1.1–1.6). These findings confirm that gallstone disease increases the risk of gallbladder cancer, whereas cholecystectomy appears to increase the risk of cancers of ampulla of Vater and pancreas. Further research is needed to clarify the carcinogenic risks associated with gallstones and cholecystectomy and to define the mechanisms involved. © 1999 Cancer Research Campaign


*British Journal of Cancer* (1999) **79**(3/4), 640–644
© 1999 Cancer Research Campaign
Article no. bjoc.1998.0101

### Gallstones, cholecystectomy and risk of cancers of the liver, biliary tract and pancreas

**WH Chow[1], C Johansen[2], G Gridley[1], L Mellemkjær[2], JH Olsen[2] and JF Fraumeni Jr[1]**

[1]Division of Cancer Epidemiology and Genetics, National Cancer Institute, Bethesda, MD, USA; [2]Danish Cancer Society, Institute of Cancer Epidemiology, Copenhagen, Denmark

**Summary** To examine the association between gallstones and cholecystectomy, we conducted a nationwide population-based cohort study in Denmark. Patients with a discharge diagnosis of gallstones from 1977 to 1989 were identified from the Danish National Registry of Patients and followed up for cancer occurrence until death or the end of 1993 by record linkage to the Danish Cancer Registry. Included in the cohort were 60 176 patients, with 471 450 person–years of follow-up. Cancer risks were estimated by standardized incidence ratios (SIRs) and 95% confidence intervals (CIs) stratified by years of follow-up and by cholecystectomy status. Among patients without cholecystectomy, the risks at 5 or more years of follow-up were significantly elevated for cancers of liver (SIR = 2.0, CI = 1.2–3.1) and gallbladder (SIR = 2.7, CI = 1.5–4.4) and near unity for cancers of extrahepatic bile duct (SIR = 1.1), ampulla of Vater (SIR = 1.0) and pancreas (SIR = 1.1). The excess risk of liver cancer was seen only among patients with a history of hepatic disease. Among cholecystectomy patients, the risks at 5 or more years of follow-up declined for cancers of liver (SIR = 1.1) and extrahepatic bile duct (SIR = 0.7), but were elevated for cancers of ampulla of Vater (SIR = 2.0, CI = 1.0–3.7) and pancreas (SIR = 1.3, CI = 1.1–1.6). These findings confirm that gallstone disease increases the risk of gallbladder cancer, whereas cholecystectomy appears to increase the risk of cancers of ampulla of Vater and pancreas. Further research is needed to clarify the carcinogenic risks associated with gallstones and cholecystectomy and to define the mechanisms involved.

**Keywords:** gallstones; cholecystectomy; neoplasms; liver; biliary tract; pancreas; cohort study

Cancers of the biliary tract encompass tumours arising from the gallbladder, extrahepatic bile ducts and ampulla of Vater. Although the aetiology of biliary tract cancers is poorly understood, an excess risk has been associated with a history of gallstone disease, especially in relation to gallbladder cancer (Lowenfels et al, 1985; Maringhini et al, 1987; Fraumeni et al, 1996). The effects of gallstones on the risk of liver and pancreas cancers, however, are unclear (Ichimiya et al, 1986; Maringhini et al, 1987; Cuzick and Babiker, 1989; Bueno de Mesquita et al, 1992; Kalapothaki et al, 1993; Ekbom et al, 1993). In a recent population-based cohort study of Swedish patients hospitalized for cholecystectomy, mostly for gallstones, the subsequent risk of extrahepatic bile duct cancer was reduced whereas the risks of periampullar and pancreatic cancers were elevated (Ekbom et al, 1993, 1996). In a previous population-based cohort study of Danish patients hospitalized for gallstones, significant excess risks were observed for cancers of the liver/biliary tract and pancreas, with the risk for pancreas cancer being greater among those treated with cholecystectomy than among non-surgical patients (Johansen et al, 1996). To pursue these findings, we examined the risk of cancers of the liver, biliary tract subsites and pancreas in an expanded nationwide population-based cohort study of Danish patients hospitalized for gallstones, stratified by cholecystectomy status.

Received 13 January 1998
Revised 6 April 1998
Accepted 22 April 1998

*Correspondence to:* WH Chow, National Cancer Institute, 6130 Executive Blvd, EPN 418, Bethesda, MD 20892-7182, USA

### MATERIALS AND METHODS

The data sources for this study have been described in detail previously (Johansen et al, 1996). The current study extended the follow-up for cancer through 1993 and used a broader cohort selection criterion to include gallstone patients with concomitant conditions such as bile duct stones, inflammation and perforation within the biliary tract. The National Registry of Patients maintains records of over 99% of hospital discharges for somatic diseases in Denmark (Danish National Board of Health, 1981). Each discharge record contained a personal identification number unique to each Danish citizen, date of discharge and up to 20 diagnoses, classified according to a Danish modification of the International Classification of Diseases, eighth revision (ICD-8). In addition, surgical procedures were recorded and classified according to the Danish Classification of Surgical Procedures and Therapies (Danish National Board of Health, 1986).

All discharge records with a diagnosis of cholelithiasis (ICD-8 574.00-09) from 1977 to 1989 were extracted. For patients with multiple hospital discharges for gallstones, the date of the first hospital discharge was used as the date of entry into the cohort. Using the personal identification number, linkage was made to the Danish National Board of Health to obtain information on vital status and to the Danish Cancer Registry for information on incident cancers (Storm et al, 1992). Of 64 420 patients initially identified from the Danish National Registry of Patients, 4244 (6.6%) were excluded because of death during the first year of follow-up, leaving 60 176 patients for the study. Patients were followed up for cancer occurrence until the date of death or the end of 1993, whichever came first. To minimize selection bias, cancer diagnoses and person–years accumulated during the first year of follow-up were excluded from analysis.

**Table 1** Standardized incidence ratios (SIRs) and 95% confidence intervals (CIs) of cancers of liver, biliary tract, and pancreas among gallstone patients by cholecystectomy status

| | Never/before cholecystectomy | | | After cholecystectomy[a] | | | Combined cohort | | |
|---|---|---|---|---|---|---|---|---|---|
| Number of subjects | | 17 715 | | | 42 461 | | | 60 176 | |
| Average age at entry | | 70 | | | 56 | | | 61 | |
| Average years at follow-up | | 6.1 | | | 9.2 | | | 8.3 | |
| Person-years | | 109 803 | | | 361 648 | | | 471 450 | |
| Type of cancer | #OBS | SIR[b] | CI | #OBS | SIR[b] | CI | #OBS | SIR[b] | CI |
| Liver | 34 | 1.9 | 1.3–2.6 | 48 | 1.2 | 0.9–1.7 | 82 | 1.4 | 1.2–1.8 |
| Gallbladder | 42 | 3.6 | 2.6–4.9 | – | – | – | 42 | 3.6 | 2.6–4.9 |
| Extrahepatic bile duct | 12 | 1.5 | 0.8–2.7 | 16 | 1.0 | 0.6–1.6 | 28 | 1.2 | 0.8–1.7 |
| Ampulla of Vater | 8 | 2.3 | 1.0–4.5 | 14 | 2.0 | 1.1–3.3 | 16 | 2.1 | 1.3–3.1 |
| Pancreas | 80 | 1.3 | 1.0–1.6 | 184 | 1.4 | 1.2–1.7 | 264 | 1.4 | 1.2–1.6 |

[a]Person–years (2308) and cancers (7) for these patients prior to their cholecystectomy were allocated to the never/before cholecystectomy stratum. [b]Adjusted for age and gender.

**Table 2** Standardized incidence ratios (SIRs) and 95% confidence intervals (CIs) of cancers of liver, biliary tract, and pancreas among gallstone patients by cholecystectomy status and years of follow-up

| | Years of follow-up | | | | | |
|---|---|---|---|---|---|---|
| | 1–4 years | | | 5+ years | | |
| | #OBS | SIRa | 95% CI | #OBS | SIRa | 95% CI |
| Never/before cholecystectomy | | | | | | |
| Liver | 14 | 1.7 | 0.9–2.8 | 20 | 2.0 | 1.2–3.1 |
| Gallbladder | 26 | 4.6 | 3.0–6.7 | 16 | 2.7 | 1.5–4.4 |
| Extrahepatic bile duct | 7 | 2.0 | 0.8–4.1 | 5 | 1.1 | 0.4–2.7 |
| Ampulla of Vater | 6 | 3.9 | 1.4–8.4 | 2 | 1.0 | 0.1–3.6 |
| Pancreas | 45 | 1.6 | 1.2–2.2 | 35 | 1.1 | 0.7–1.5 |
| After cholecystectomy[b] | | | | | | |
| Liver | 20 | 1.6 | 1.0–2.5 | 28 | 1.1 | 0.7–1.5 |
| Extrahepatic bile duct | 8 | 1.6 | 0.7–3.2 | 8 | 0.7 | 0.3–1.4 |
| Ampulla of Vater | 4 | 1.9 | 0.5–4.8 | 10 | 2.0 | 1.0–3.7 |
| Pancreas | 72 | 1.8 | 1.4–2.2 | 112 | 1.3 | 1.1–1.6 |

[a]Adjusted for age and gender. [b]Person–years for this stratum were accumulated after the cholecystectomy which might be subsequent to cohort entry.

Among the cohort members, 42 461 (65.9%) were treated by cholecystectomy, 96% of operations being performed within 6 months of the index hospitalization for gallstones. Analyses were conducted for the entire cohort, and with stratification by cholecystectomy status. Person–years and cancer incidence before cholecystectomy were classified with the non-cholecystectomy stratum, and person–years for the cholecystectomy stratum were accumulated following the surgery. Within the cholecystectomy subcohort, further analyses were conducted to examine whether risks varied with or without accompanying bile duct surgery, which may affect risk by associated bile duct stones or subsequent alterations in bile flow. Furthermore, to assess whether risks were confounded by other conditions associated with gallstone disease and the cancer outcomes of interest, additional analyses were conducted with stratification by obesity and by selected hepatic diseases, including hepatitis, cirrhosis, haemochromatosis and alcoholism, as discharge diagnoses during any hospital visit.

The expected numbers of cancers were calculated by multiplying the number of person–years in the cohort by the national cancer incidence rates for each stratum by sex and age in 5-year groups. Standardized incidence ratios (SIRs), the ratio of observed to expected cancer cases and corresponding 95% confidence intervals (CIs) were computed for 1–4 and ≥5 years intervals following hospital discharge for gallstones. Tests of significance for the SIR were based on the assumption that the observed number of a specific cancer followed a Poisson distribution, with a mean value equal to the expected number derived from the general population (Bailar and Ederer, 1964). In addition, to assess the potential for surveillance bias, SIRs were computed after excluding patients whose cancer of interest was diagnosed incidentally during autopsy from both the observed number as well as from the population rates used in calculating the expected number. Biliary tract cancer risk was evaluated by anatomical subsite, including the gallbladder, extrahepatic bile ducts and ampulla of Vater. For comparison, SIRs were computed for cancers arising from adjacent sites, i.e. the liver and pancreas.

© Cancer Research Campaign 1999

**Table 3** Standardized incidence ratios (SIRs) of cancers of liver, biliary tract and pancreas among gallstone patients with stratification by cohort characteristics

| | Liver | | Gallbladder | | Extrahepatic bile duct | | Ampulla of Vater | | Pancreas | |
|---|---|---|---|---|---|---|---|---|---|---|
| Stratification variable | #OBS | SIR | #OBS | SIR | #OBS | SIR | #OBS | SIR | #OBS | SIR |
| Bile duct surgery[a] | | | | | | | | | | |
| No (PY[b] = 282 195) | 39 | 1.5[c] | NA[d] | – | 11 | 1.0 | 10 | 2.0 | 110 | 1.2[c] |
| Yes (PY = 79 453) | 9 | 0.8 | NA | – | 5 | 1.0 | 4 | 1.8 | 74 | 1.9[c] |
| Obesity | | | | | | | | | | |
| No (PY = 447 911) | 77 | 1.4[c] | 39 | 3.6[c] | 28 | 1.2 | 19 | 1.9[c] | 245 | 1.4[c] |
| Yes (PY = 23 539) | 5 | 2.1 | 3 | 3.6 | 0 | 0.0 | 3 | 6.5[c] | 19 | 2.2[c] |
| Hepatic diseases[e] | | | | | | | | | | |
| No (PY = 455 253) | 53 | 1.0 | 40 | 3.5[c] | 27 | 1.2 | 20 | 1.9[c] | 252 | 1.4[c] |
| Yes (PY = 16 197) | 29 | 15.0[c] | 2 | 6.0 | 1 | 1.4 | 2 | 6.2 | 12 | 2.0[c] |
| Excluded autopsy | | | | | | | | | | |
| Never/before cholecystectomy | 17 | 1.2 | 37 | 3.7[c] | 12 | 1.6 | 4 | 1.2 | 70 | 1.3 |
| After cholecystectomy | 42 | 1.4 | NA | – | 16 | 1.0 | 14 | 2.1[c] | 168 | 1.4[c] |

[a]Cholecystectomy with accompanying bile duct surgery; excluded non-surgical patients. [b]Person–years. [c]$P < 0.05$. [d]Not applicable. [e]Included hepatitis, cirrhosis, haemochromatosis and alcoholism.

### RESULTS

A total of 471 450 person–years of follow-up were accrued, with 109 803 person–years allocated in the non-cholecystectomy stratum and 361 648 in the cholecystectomy stratum (Table 1). The non-cholecystectomy group was much older at cohort entry than the cholecystectomy group (mean age 70 vs 56 years). There were generally more women than men, with a female-to-male ratio of 2.1 in the non-cholecystectomy group and 2.8 in the cholecystectomy group. Among those without cholecystectomy, the mean duration of follow-up was 6.1 years after cohort entry versus 9.2 years following cholecystectomy.

Overall, the risks for liver and extrahepatic bile duct tumours tended to be higher in the non-cholecystectomy than the cholecystectomy group, although these differences were not statistically significant (Table 1). Risk estimates for the non-cholecystectomy stratum were essentially unaltered when person–years (2308) and cancers (7) for patients who subsequently had cholecystectomy were excluded (data not shown).

In the non-cholecystectomy group, the risks for cancers of liver and gallbladder remained significantly elevated at 5 or more years of follow-up (SIR = 2.0 and 2.7 respectively), as shown in Table 2. In contrast, the risks for cancers of extrahepatic bile duct, ampulla of Vater and pancreas declined to near unity at 5 or more years of follow-up.

On the other hand, in the cholecystectomy group, the risks for cancers of liver and extrahepatic bile duct cancers dropped to 1.1 and 0.7, respectively, at 5 or more years of follow-up. In contrast, the risks for cancers of ampulla of Vater and pancreas remained elevated (SIR = 2.0 and 1.3 respectively), with the excess of pancreas cancer being statistically significant throughout follow-up.

In the cholecystectomy stratum, the risks for cancers of extrahepatic bile duct and ampulla of Vater were similar among patients who had cholecystectomy only and those who had bile duct surgery along with cholecystectomy (Table 3). However, the risk for liver cancer was higher among patients with cholecystectomy only than among those who also had bile duct surgery, whereas the risk for pancreas cancer was higher among those with accompanying bile duct surgery than among those with cholecystectomy only.

The cancer risks (except for liver cancer) were essentially unaltered when the analyses were restricted to patients who never had a hospital discharge diagnosis of obesity or hepatic diseases (Table 3). For liver cancer, however, the risks were near unity among those without a history of hepatic conditions and were increased substantially among those with such a history. The results were similar regardless of cholecystectomy status (data not shown). The numbers of biliary tract cancers among patients with a diagnosis of obesity or hepatic conditions were too small for meaningful analysis, whereas the risk for pancreas cancer was increased twofold.

Exclusion of cancer cases diagnosed incidentally during autopsy did not appreciably alter the associations seen for cancers of gallbladder, extrahepatic bile duct, ampulla of Vater and pancreas (Table 3). The risk of liver cancer, however, was reduced to 1.2 among those without cholecystectomy, but remained slightly elevated at 1.4 among those treated with cholecystectomy.

### DISCUSSION

Our findings among gallstone patients without cholecystectomy are consistent with previous reports indicating that gallstone disease predisposes to biliary tract cancers, particularly gallbladder cancer (Lowenfels et al, 1985; Maringhini et al, 1987; Yen et al, 1987; Chow et al, 1994; Fraumeni et al, 1996). Although the risk for gallbladder cancer remained elevated at 5 or more years after hospitalization for gallstone disease, the risks for cancers of extrahepatic bile duct and ampulla of Vater dropped to near unity, suggesting that residual selection bias associated with the detection of existing tumours during the early years of follow-up contributed to the overall excess of bile duct and ampullary tumours. The risk for extrahepatic bile duct cancers tended to be lower among patients with cholecystectomy than among those not treated surgically, with risk dropping to below unity at 5 or more years of follow-up. This finding is consistent with an earlier Swedish cohort study of cholecystectomy patients indicating a significantly lowered risk of extrahepatic bile duct cancer after long-term follow-up (Ekbom et al, 1993).

Also consistent with the recent Swedish study (Ekbom et al, 1996), patients in our cholecystectomy group had significantly

© Cancer Research Campaign 1999

elevated risks for cancers of ampulla of Vater and pancreas that persisted after 5 or more years of follow-up, although the long-term excess of pancreatic cancer was small. While selection bias, small numbers and chance variation may partly explain this finding, it seems plausible that cholecystectomy rather than gallstone formation is the more important risk factor for cancers of ampulla of Vater and perhaps pancreas. In laboratory animals, cholecystectomy has been shown to stimulate pancreatic hypertrophy and hyperplasia (Rosenberg et al, 1984) and predispose to benign and malignant tumours of the pancreas (Ura et al, 1986; Watanapa and Williamson, 1993). Although the mechanism of action is unclear, it has been suggested that cholecystectomy increases circulating levels of cholecystokinin–pancreozymin, a gastrointestinal hormone with trophic effects on the pancreas (Watanapa and Williamson, 1993; Anderson et al, 1996).

The elevated risk of liver cancer among patients without cholecystectomy may be explained, in part, by increased surveillance, as exclusion of cases diagnosed incidentally at autopsy reduced the risk to near unity. In addition, the risk of liver cancer was reduced to unity among cohort members who were not previously hospitalized for hepatic diseases, suggesting that gallstone disease without hepatic complications is not an important risk factor for liver cancer. As gallstones and other extrahepatic diseases may increase inflammatory mediators such as cytokine expression in the liver (Fukuma et al, 1996), however, further assessment of the risk of liver cancer among gallstone patients may be warranted.

While obesity is a well-established risk factor for gallbladder cancer (Fraumeni et al, 1996) and may also be linked to cancers of liver and pancreas (Moller et al, 1994), exclusion of patients who were never hospitalized with obesity did not affect the risk estimates. These findings suggest that obesity is unlikely to account for the entire excesses of these tumours in our cohort.

A few methodological issues should be considered in interpreting findings from our registry-based cohort study. Reporting of cancers diagnosed among Danish residents and follow-up of cohort members for death and emigration are nearly complete (Storm et al, 1992), thus minimizing selection bias. In Denmark, nearly all diagnoses of gallstones are verified by radiography or ultrasonography, so that misclassification of non-cases into the cohort is unlikely. However, a substantial proportion of individuals with gallstones may remain undiagnosed. To the extent that patients hospitalized for gallstones may have risk patterns that differ from those of individuals with 'silent' stones or mild symptoms not requiring hospitalization, our findings are not generalizable to non-hospitalized patients. Furthermore, if a substantial proportion of the general population has undiagnosed gallstones which predispose to cancer, then risks in our cohort may be underestimated. On the other hand, it is possible that cholecystectomies were sometimes carried out on account of the early symptoms of biliary tract cancers which had been erroneously attributed to the gallstones that were present. This may have contributed to the excess of certain biliary tract cancers, at least in the early period. To minimize bias due to diagnosis of prevalent cancers, we excluded the person–years and cancers occurring during the first year of follow-up. Although some of the significant results in our study might have occurred by chance because of multiple comparisons, the consistency of the risk patterns within our dataset and with results of the Swedish study supports the validity of our findings.

Information on potential confounding factors such as cigarette smoking and alcohol drinking was not available for adjustment. In our cohort, however, there was no excess risk for cancers that are strongly linked to use of tobacco or alcohol, such as lung cancer (SIR = 1.0, 95% CI = 0.9–1.1) and cancers of the buccal cavity and pharynx (SIR = 1.1, 95% CI = 0.9–1.4), suggesting that these risk factors are unlikely to have influenced our findings. It should be noted that the results for patients with and without cholecystectomy may not be directly comparable, as the non-surgical patients were much older and likely to have other medical conditions that precluded surgery.

In conclusion, patients hospitalized for gallstones had an elevated risk of subsequent cancers of the liver, biliary tract and pancreas. Treatment with cholecystectomy was associated with a reduction in the long-term risk for cancers of liver and extrahepatic bile ducts, but not for cancers of ampulla of Vater and pancreas. Further research is needed to clarify the carcinogenic risks associated with gallstones and cholecystectomy and to define the mechanisms involved.

### ACKNOWLEDGEMENTS

The authors thank Ms Shannon Keehn of IMS, Inc. and Ms Andrea Bautz of the Danish Cancer Society, Institute of Cancer Epidemiology for computing support.

### REFERENCES

Anderson KE, Potter JD and Mack TM (1996) Pancreatic cancer. In *Cancer Epidemiology and Prevention*, 2nd edn, Schottenfeld D and Fraumeni JF Jr (eds) pp. 725–771. Oxford University Press: New York

Bailar JC and Ederer F (1964) Significance factors for the ratio of a Poisson variable to its expectation. *Biometrics* **20**: 639–643

Bueno de Mesquita HB, Maisonneuve P, Moerman CJ and Walker AM (1992) Aspects of medical history and exocrine carcinoma of the pancreas: a population-based case–control study in The Netherlands. *Int J Cancer* **52**: 17–23

Chow WH, McLaughlin JK, Menck HR and Mack TM (1994) Risk factors for extrahepatic bile duct cancers: Los Angeles County, California (USA). *Cancer Causes Control* **5**: 267–272

Cuzick J and Babiker AG (1989) Pancreatic cancer, alcohol, diabetes mellitus and gall-bladder disease. *Int J Cancer* **43**: 415–421

Danish National Board of Health (1981) *The Activity in the Hospital Care System (in Danish)*. Danish National Board of Health: Copenhagen

Danish National Board of Health (1986) *Classification of Diseases (1976) (in Danish)*. Danish National Board of Health: Copenhagen

Ekbom A, Hsieh C-C, Yuen J, Trichopoulos D, McLaughlin JK, Lan S-J and Adami H-O (1993) Risk of extrahepatic bileduct cancer after cholecystectomy. *Lancet* **342**: 1262–1265

Ekbom A, Yuen J, Karlsson BM, McLaughlin JK and Adami H-O (1996) Risk of pancreatic and periampullar cancer following cholecystectomy: a population-based cohort study. *Dig Dis Sci* **41**: 387–391

Fraumeni JF Jr, Devesa SS, McLaughlin JK and Stanford JL (1996) Biliary tract cancer. In *Cancer Epidemiology and Prevention*, 2nd edn, Schottenfeld D and Fraumeni JF Jr. (eds), pp. 794–805. Oxford University Press: New York

Fukuma H, Morshed A, Watanabe S, Uchida N, Ezaki T, Minami A, Matsuoka H, Hirabayashi S, Nakatsu T and Nishioka M (1996) Increased expression of cytokines in liver and serum in patients with extrahepatic diseases. *J Gastroenterol* **31**: 538–545

Ichimiya H, Kono S, Ikeda M, Tokudome S, Nakayama F and Kuratsune M (1986) Cancer mortality among patients undergoing cholecystectomy for benign biliary diseases. *Jpn J Cancer Res* **77**: 579–583

Johansen C, Chow WH, Jørgensen T, Mellemkjær L, Engholm G and Olsen JH (1996) Risk of colorectal cancer and other cancers in patients with gall stones. *Gut* **39**: 439–443

Kalapothaki V, Tzonou A, Hsieh CC, Toupadaki N, Karakatsani A and Trichopoulos D (1993) Tobacco, ethanol, coffee, pancreatitis, diabetes mellitus, and cholelithiasis as risk factors for pancreatic carcinoma. *Cancer Causes Control* **4**: 375–382

Lowenfels AB, Lindström CG, Conway MJ and Hastings PR (1985) Gallstones and risk of gallbladder cancer. *J Natl Cancer Inst* **75**: 77–80

© Cancer Research Campaign 1999

Maringhini A, Moreau JA, Melton J III, Hench VS, Zinsmeister AR and DiMagno EP (1987) Gallstones, gallbladder cancer, and other gastrointestinal malignancies: an epidemiologic study in Rochester, Minnesota. *Ann Intern Med* **107**: 30–35

Moller H, Mellemgaard A, Lindvig K and Olsen JH (1994) Obesity and cancer risk: a Danish record-linkage study. *Eur J Cancer* **30A**: 344–350

Rosenberg L, Duguid WP and Brown RA (1984) Cholecystectomy stimulates hypertrophy and hyperplasia in the hamster pancreas. *J Surg Res* **37**: 108–111

Storm HH, Manders T, Friis S and Band S (1992) *Cancer Incidence in Denmark 1989*. Danish Cancer Society: Copenhagen

Ura H, Makino T, Ito S, Tsutsumi M, Kinugasa T, Kamano T, Ichimiya H and Konishi Y (1986) Combined effects of cholecystectomy and lithocholic acid on pancreatic carcinogenesis of N-nitroso-bis(2-hydroxypropyl)amine in Syrian golden hamsters. *Cancer Res* **46**: 4782–4786

Watanapa P and Williamson RCN (1993) Experimental pancreatic hyperplasia and neoplasia: effects of dietary and surgical manipulation. *Br J Cancer* **67**: 877–884

Yen S, Hsieh C-C and MacMahon B (1987) Extrahepatic bile duct cancer and smoking, beverage consumption, past medical history, and oral-contraceptive use. *Cancer* **59**: 2112–2116

*© Cancer Research Campaign 1999*
